# A platform in the use of medicines to treat chronic hepatitis C (PLATINUM C): protocol for a prospective treatment registry of real-world outcomes for hepatitis C

**DOI:** 10.1186/s12879-020-05531-4

**Published:** 2020-10-29

**Authors:** Jessica Ramsay, Julie Marsh, Alisa Pedrana, Nada Andric, Richard Norman, Wendy Cheng, Steve Webb, Nikolajs Zeps, Matthew Bellgard, Todd Graves, Margaret Hellard, Tom Snelling

**Affiliations:** 1 Wesfarmers Centre of Vaccines and Infectious Diseases, Telethon Kids Institute, The University of Western Australia, Perth, Australia; 2 Disease Elimination Program, Burnet Institute, Melbourne, Australia; 3 School of Public Health and Preventive Medicine, Monash University, Melbourne, Australia; 4 Homeless Healthcare, West Leederville, Perth, Australia; 5 School of Public Health, Curtin University, Bentley, Australia; 6 Department of Gastroenterology and Hepatology, Royal Perth Hospital, Perth, Australia; 7 UWA Medical School, University of Western Australia, Perth, Australia; 8 School of Medical and Health Sciences, Edith Cowan University, Perth, Australia; 9 St John of God Hospital, Subiaco, Perth, Australia; 10 Epworth HealthCare, Eastern Clinical School of Monash University, Melbourne, Australia; 11 eResearch Office, Queensland University of Technology, Brisbane, Australia; 12 Berry Consultants, Austin, TX USA; 13 Department of Infectious Diseases, The Alfred and Monash University, Melbourne, Australia; 14 Peter Doherty Institute for Infection and Immunity, Melbourne, Australia; 15 School of Population and Global Health, University of Melbourne, Melbourne, Australia; 16 Menzies School of Health Research, Charles Darwin University, Darwin, Australia; 17 Department of Infectious Diseases, Perth Children’s Hospital, Perth, Australia; 18 School of Public Health, University of Sydney, Camperdown, Sydney, New South Wales 2006 Australia

**Keywords:** Hepatitis C, Direct-acting antiviral treatment, Treatment registry, Platform trial

## Abstract

**Background:**

Safe, highly curative, short course, direct acting antiviral (DAA) therapies are now available to treat chronic hepatitis C. DAA therapy is freely available to all adults chronically infected with the hepatitis C virus (HCV) in Australia. If left untreated, hepatitis C may lead to progressive hepatic fibrosis, cirrhosis and hepatocellular carcinoma. Australia is committed to eliminating hepatitis as a public health threat by 2030 set by the World Health Organization. However, since the introduction of funded DAA treatment, uptake has been suboptimal. Australia needs improved strategies for testing, treatment uptake and treatment completion to address the persisting hepatitis C public health problem. PLATINUM C is a HCV treatment registry and research platform for assessing the comparative effectiveness of alternative interventions for achieving virological cure.

**Methods:**

PLATINUM C will prospectively enrol people with active HCV infection confirmed by recent detection of HCV ribonucleic acid (RNA) in blood. Those enrolled will agree to allow standardised collection of demographic, lifestyle, treatment, virological outcome and other relevant clinical data to better inform the future management of HCV infection. The primary outcome is virological cure evidenced by sustained virological response (SVR), which is defined as a negative HCV PCR result 6 to 18 months after initial prescription of DAA therapy and no less than 12 weeks after the completion of treatment. Study participants will be invited to opt-in to medication adherence monitoring and quality of life assessments using validated self-reported instruments (EQ-5D-5L).

**Discussion:**

PLATINUM C is a treatment registry and platform for nesting pragmatic trials. Data collected will inform the design, development and implementation of pragmatic trials. The digital infrastructure, study procedures and governing systems established by the registry will allow PLATINUM C to support a wider research platform in the management of hepatitis C in primary care.

**Trial registration:**

The trial is registered with the Australia and New Zealand Clinical Trials Register (ACTRN12619000023156). Date of registration: 10/01/2019.

**Supplementary Information:**

**Supplementary information** accompanies this paper at 10.1186/s12879-020-05531-4.

## Background

### Hepatitis C in Australia

Hepatitis C virus (HCV) infection is a major public health issue in Australia and other countries due to the substantial morbidity associated with chronic infection. It is estimated 71 million people are living with HCV worldwide including over 180,000 in Australia [1, 2]. Those chronically infected with HCV, if left untreated, are at risk of progressive hepatic fibrosis, cirrhosis and hepatocellular carcinoma.

With the recent development of direct acting antiviral (DAA) therapy, HCV infection is now curable in the majority of those infected with drugs that are safe, well tolerated, and highly effective. In response the World Health Organization called for the elimination of hepatitis C as a public health threat and set targets to reduce HCV-related mortality by 65% and HCV infection incidence by 80% by 2030 [3].

Australia was one of the first countries globally to make treatment available for all. From March 2016, all adults chronically infected with HCV in Australia have been eligible for treatment with DAA funded by the Government under the Pharmaceutical Benefit Scheme (PBS). Implementation of DAA therapies has led to a shift of HCV management from specialised tertiary care to the primary care setting. Initially, prescribing the newly licenced DAAs was complicated by the number of drugs available and treatment guidelines that factor in virus genotype, cirrhosis status, previous treatment status, drug-drug interactions and presence of comorbidities.

If the WHO elimination targets are to be met, effective strategies are needed to increase the number of people being tested, treated and cured [4]. These strategies are still largely unknown. Those most affected by HCV infection are often those who are relatively under-served by, or who have difficulties accessing, health care [5]. This includes people who inject drugs, incarcerated populations, people in rural and remote settings, Indigenous and migrant populations [6, 7].

### Aim

The overall aim of the PLATINUM C treatment registry is to inform the improved management of HCV in primary care. It is envisioned that this will provide a research platform to enable the embedding of pragmatic trials. This protocol outlines the background, objectives, endpoints, data collection and study procedures generic to this research platform and sets out the purpose and rational for establishment of the treatment registry.

## Methods/design

### Study design

This study is a prospective, multi-site treatment registry of primary care practices within Australia. Individual participation in the PLATINUM C research platform involves the capture of consenting participants’ demographic and clinical data and HCV treatment and outcome data. This information will be used to compare treatment response rates in different subgroups of patients. Where important variation in practice and outcomes are identified, this data will be used, as part of a research prioritisation process, in consultation with the Platinum C Community Reference Group to inform the design, development and implementation of pragmatic trials involving randomised assignment of alternative treatment options.

### Study population and setting

PLATINUM C will enrol adults (≥ 18 years) who have confirmed active HCV infection (positive HCV RNA PCR within 3 months) from community primary health care clinics which are involved in delivering HCV infection care. Ethical approval has been obtained for 5 high caseload primary health care clinics across metropolitan Perth, Western Australia, where the underlining chronic HCV prevalence ranges between 0.66–0.68%. Additional study sites across Australia, where the national prevalence was estimated to be 0.78% at the start of 2016, will be included with ethical permission as the study progresses [8]. Participating clinics will agree to comply with study procedures including the embedding of the data collection and prescribing tools within their clinical practice.

### Objectives

The primary objective of PLATINUM C is, for adults with chronic HCV infection, to determine the absolute and comparative effectiveness of a range of interventions to achieve a HCV virological cure. This is demonstrated by a sustained viral response (SVR), defined as an undetectable HCV ribonucleic acid (RNA) ≥12 weeks after discontinuation of antiviral treatment and within 18 months of the start of treatment. Interventions will include licenced and funded DAA therapies and other management strategies to be defined by platform embedded trials.

The secondary objectives are to describe chronic HCV management in primary care for different patient subgroups, including by: (i) describing the distribution of the treatment response under alternative management options, (ii) characterising treatment adherence, (iii) identifying demographic and clinical characteristics associated with high risk of lost-to-follow-up from standard care, and (iv) describing and comparing patterns of health care utilisation.

### Outcomes

The primary outcome is virological cure as evidenced by the endpoint of sustained virological response (SVR) defined as a negative HCV RNA (in plasma) PCR result 6 to 18 months after the initial prescription of DAA therapy and no less than 12 weeks after the end of antiviral treatment.

Secondary outcomes and endpoints are:

(i) Completion of follow up defined by the completion of SVR12 testing (HCV RNA PCR)
(ii) Adherence of study participants to antiviral treatment recorded as the self-reported proportion of total prescribed doses taken,
(iii) Quality of life, as measured by the self-reported EQ-5D-5L questionnaire administered during and for up to 24 months after initiation of treatment and,
(iv) Healthcare utilisation defined as any attendance for healthcare related service that attracts a cost to the individual, insurer or government.

### Study duration

There is no specified end date for the research platform. It is anticipated that PLATINUM C will continue to enrol participants and integrate the testing of new interventions and/or groups of interventions until one of the following occurs:

- The effectiveness and/or cost-effectiveness of all possible interventions are known and there are no new interventions to evaluate
- Chronic HCV infection is no longer deemed to be a public health problem
- Funding or other necessary support is no longer available

Should the platform be stopped, the end of this study is the date of the last scheduled follow up for any participant.

### Study procedures

#### Informed consent

A participant must provide informed consent (written or electronically) before any specific procedures are performed or data collected. Electronic and/or written versions of the participant information sheet will be presented to the participant detailing no less than: the exact nature of the project; what it will involve for the participant; the implications and constraints of the protocol; any risks involved in taking part. It will be clearly stated that participation is voluntary.

#### Withdrawal of participants

Participants have the right to withdraw from this study at any time without penalty or loss of benefits. Data obtained prior to participants’ withdrawal from the study will be included in the analysis.

#### Data collection

Relevant data (outlined in Table 1) will be obtained directly from the healthcare providers and participants. Data will be collected through data capture and prescribing tools and phone and/or email participant surveys.

**Table 1:** Summary of data collection

Data type	Information recorded	Time point
Demographics	Age, gender, postcode, Indigenous status	Enrolment
Lifestyle	Self-reported injecting drug use, self-reported alcohol in-take, self-reported housing status, self-reported reuse of a needle/syringe, self-reported prescribed opioid substitution therapy, risk of treatment disruption (including imprisonment)	Enrolment
Clinical	Hepatitis C history, previous treatment, comorbidities, HCV genotype and RNA level, liver function tests, liver fibrosis assessment	Treatment prescription
Treatment	DAA therapy and duration prescribed, treatment setting, type of prescriber, concurrent medication, other concurrent HCV management interventions	Treatment prescription
Medication adherence	Self-reported adherence to therapy	Weekly during treatment course
Quality of life	Self-reported quality of life (QoL) score (EQ-5D-5L)	0, 3, 6, 12 and 24 months after treatment initiation
Outcomes	HCV RNA PCR, all re-collected laboratory and imaging results, self-reported injecting drug use,	3 months after treatment completion

**Fig. 1:**
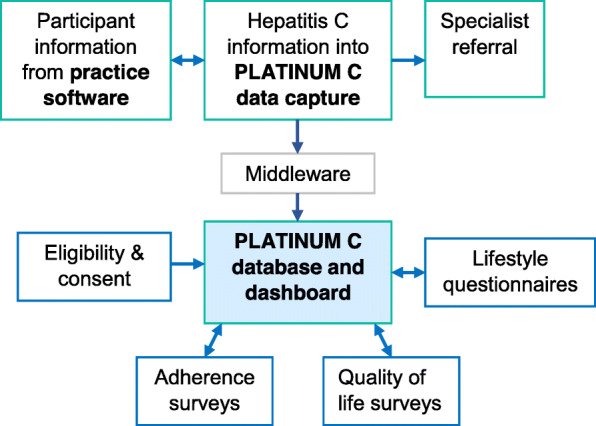
Study workflow and utilisation of the data capture tool

PLATINUM C will use a direct data capture tool that is integrated with the practice software (described in Fig. 1). Data from consenting participants will be extracted and uploaded to the data capture system. Information unable to be extracted with be uploaded by the consulting practitioner. The data capture tool will ensure all information required for prescribing DAAs or referral for specialist consultation is collected. The data capture tool will also serve to provide prescribing recommendation based on the Australia recommendation for genotype, cirrhosis level and treatment experience. Patient information collected will be stored within the practice software and de-identified participant information will be fed directly into a secure online database via secure middleware. A monitoring dashboard will allow healthcare staff to track when participants are due for follow-up SVR12 testing and long-term cirrhosis assessments.

Data will be stored on a password protected electronic database housed securely on a server at the Telethon Kids Institute in Perth, Western Australia, and will be archived until 15 years after the end of enrolment or publication, whichever is later.

##### Baseline and clinical outcome assessment

Prospective baseline, treatment and outcome data collection will be integrated into standard clinical care. This information is currently routinely required in the management of hepatitis C in Australia as per standard guidelines from the Gastroenterological Society of Australia and Australian Liver Association [9]. Embedding of data collection of clinical information will be facilitated by a HCV prescribing tool.

##### Life-style questionnaire

Participants will be asked to complete a questionnaire (see Additional file 1) to further characterise risk behaviours, treatment responses and healthcare utilisation across different patient groups including among marginalised groups who are traditionally underserved by the healthcare system. These questionnaires were developed in consultation with the Platinum C Community Reference Group.

##### Adherence monitoring

Participants will be invited to self-report medication adherence. Upon initiation of HCV antiviral therapy, participants who opt-in will receive either a weekly phone or text message asking them to provide the number of doses taken in the preceding 7 days.

##### Quality of life questionnaire

The self-reported EQ-5D-5L quality of life instrument [10] will be issued electronically to those who opt-in at 0, 3, 6, 12 and 24 months after initiation of DAA therapy.

#### Data analysis

Primary analyses of all data will use the intention-to-treat (ITT) principle. Demographic, clinical and other baseline variables will be summarised by viral genotype, liver cirrhosis status, previous HCV treatment and clinician prescribed treatment. Continuous variables will be summarised as mean and standard deviation for symmetric distributions and median and interquartile range (IQR) for asymmetric distributions. Categorical variables will be summarised at each level as frequency and percentage. Frequencies below five will be reported as “< 5” to protect individual confidentiality.

For the primary endpoint a Bayesian logistic regression model, with weakly informative priors, will be used to compare treatment groups and incorporate Bayesian borrowing across pre-specified subgroups. The exact model will depend on the trial modules (and interventions) defined and will be documented in the statistical analysis plan (SAP). The proportion of participants that achieve SVR12 will be reported every 200 participants. Secondary endpoints will be similarly summarised and analysed using Bayesian regression models, which will be documented in the SAP.

Self-reported DAA treatment adherence will be summarised as the median (and IQR) proportion of prescribed doses taken in weeks 1 to 12 by viral genotype, liver cirrhosis status and clinician prescribed treatment. Self-reported quality of life measured during and for up to 24 months after initiation of treatment, as measured by the EQ-5D-5L, will be summarised by median (and IQR) by viral genotype, liver cirrhosis status and clinician prescribed treatment.

#### Community and stakeholder involvement

Community and stakeholder involvement has been central to the development of this research platform and will continue to serve an important role. Regular consultation of the Platinum C stakeholder advisory committee and community reference group will help ensure all procedures, aims and outcomes are of relevance and importance to those affected by HCV.

#### Limitations

While we expect engagement with primary care practices will increase participation in the PLATINUM C registry, we are limited in our ability to capture all potential participants presenting to study sites. It is likely some individuals will be treated without being invited to participate and others will decline. However, the digital infrastructure created for PLATINUM C aims to make it easier for prescribers to manage HCV.

It is likely that some participants will be lost-to-follow-up, thereby making outcome information unobtainable. This issue has been well-described within the HCV-infected population [11] and identified as a risk by our community and stakeholder consultation. As information is collected from both prescribers and patients we anticipate this registry will mitigate this risk by directly engaging people infected with HCV and establishing secure data collection.

#### Future directions

Studies of individual interventions or complex strategies to better implement DAA therapy are an area of active research. A limitation of conventional clinical trials is that even if an intervention is proven effective, it may not be possible to know whether the benefit of an individual intervention is transferable to other contexts. Also, it can be difficult to identify which element(s) of a complex intervention are effective (or cost-effective) and which may be deleterious, especially if implemented in different treatment settings. Such trials are inefficient for identifying treatment responsive patient subgroups or for identifying combinations of interventions which are synergistic or antagonistic. A platform trial embedded within the PLATINUM C treatment registry could help in the evaluation of strategies to optimise the number of people infected with HCV infection engaging with care, receiving treatment, and being cured. A large amount of information is required to adequately design, plan and implement platform trials, ensuring populations with the highest burden are adequately represented. PLATINUM C will do this by implementing a prospective HCV treatment registry using digital data collection and prescribing tools embedded in primary health care.

## Discussion

Safe, highly effective, short course, DAA therapies are now available to treat chronic HCV infection. Despite being subsidised by the Australian Government since 2015, it was estimated that only 33% of those infected had been treated by the end 2018 [12, 13]. To achieve elimination, strategies are required to increase testing, treatment uptake and treatment completion in primary care settings. There are several time and cost barriers that inhibit embedding of traditional trials within primary care. We have set out to develop a digital HCV treatment registry to facilitate DAA prescribing in primary care, while providing a research platform for nesting pragmatic clinical trials.

Randomised Embedded Multi-Arm Pragmatic (REMAP) platform trials offer a new approach to optimising the management of complex conditions [14, 15]; however, they are complex and require accurate, timely and efficient collection of data. With successful implementation and integration into routine healthcare, such trials offer many advantages over traditional clinical trials. Multiple interventions may be evaluated across different subgroups of patients. This novel trial approach is suited to HCV management in Australia due to the evolving disease landscape, numerous new treatment and interventions, the heterogenous disease populations and Australia’s commitment to disease elimination by 2030.

Any trials embedded within this platform will allow participants to opt in to receive treatment which is randomly assigned. This will be achieved by extending this core protocol through the addition of modules which will cover all additional procedures for the random assignment of patients to various treatment options, including the capture of adverse events and statistical analyses. Consistent with the overall objective of PLATINUM C, all additions and adaptations will evaluate the real-world effectiveness of HCV treatments and interventions which aim to achieve virological cure. Implementation of these trials will be guided by the information collected within the treatment registry characterising both the patient and prescriber population.

Ensuring quality study procedures such as effective data collection and obtaining valid informed consent is of utmost importance for vulnerable participant populations participating in complex study settings. PLATINUM C has been designed to establish the digital infrastructure needed to allow evaluation of alternative interventions and can therefore accommodate both observational and randomised, controlled comparative effectiveness studies. Eligibility is intended to be inclusive for all individuals eligible for hepatitis C direct acting antiviral therapy. The creation of digital infrastructure to support registry-nested trials will facilitate efficient standardised data collection including capture of endpoints. Shared oversight of the platform by a joint investigator-consumer steering committee is expected to support a learning health systems approach to hepatitis C management in primary care.

## Supplementary Information

**Additional file 1.** Life-style questionnaire.

## Data Availability

The datasets used and/or analysed during this study will be available for research purposes from the corresponding author upon reasonable requests.

## Abbreviations

DAA: direct acting antivirals
EQ-5D-5L: EuroQol -5D-5L
HCV: hepatitis C virus
IQR: interquartile range
PBS: Pharmaceutical Benefit Scheme
PCR: polymerase chain reaction
RNA: ribonucleic acid
SAP: statistical analysis plan
SVR: sustained virological response
