# Supplementary material for: A platform in the use of medicines to treat chronic hepatitis C (PLATINUM C): protocol for a prospective treatment registry of real-world outcomes for hepatitis C
Source: BMC Infect Dis. 2020 Oct 29;20:802. doi: 10.1186/s12879-020-05531-4 (PMC7596998; doi:10.1186/s12879-020-05531-4)
Supplement: Supplementary file 1 — Additional file 1. Life-style questionnaire. [file 12879_2020_5531_MOESM1_ESM.docx]

Additional file 1 – Life style questionnaire

| Platinum C Study Questionnaire | |
| --- | --- |
| What is your housing situation today? | **I do not have housing (I am staying with others, in a hotel, in a shelter, living outside on the street, on a beach, in a car, abandoned building, bus or train station, or in a park)**  **I have housing today, but I am worried about losing housing in the future**  **I have housing** |
| Why are we asking this?  Moving around may make it harder for you to collect, keep and take your hepatitis C tablets every day. And you might move away from your normal pharmacy.  Knowing this can help your healthcare practitioner make it easier for you. For example, they can organise for the pharmacy to store your tablets, or set up a way to help move your medicines with you. | |
| In a normal week, how many drinks do you have per day  how often per week do you drink? | **none**  **I used to drink, but now I don’t drink anymore**   - **I used to drink ____ standard drinks/day** - **I used to drink ____ days/week**   **Yes**   - **I drink ___ standard drinks/day** - **I drink ___days/week** |
| Why are we asking this?  Both alcohol and hepatitis C can damage your liver. If you drink a lot of alcohol or have in the past, you may have additional liver damage. This may require monitoring during and after your hepatitis C treatment. So, it is helpful to know how much you drink or drank in the past.  While drinking alcohol, it can also be difficult to remember to take your tablets regularly. If your healthcare practitioner know knows this, they can offer some options that may help you to remember to take your medications every day.  What is a standard drink?  1 standard drink = 10g alcohol   - 285ml (middy) of ‘heavy’ beer - 375ml study ‘mid strength’ beer - 120ml glass of table wine - 60ml glass of port sherry - 30ml nip of spirits | |
| Have you ever injected drugs? | **No, Never**  **Not in the past 3 months**  **Yes, within the past 3 months** |
| Why are we asking this?  Studies have shown that people who are currently injecting drugs are the most important people to be treated for hepatitis C.  If you tell us about your injecting drug use, your healthcare practitioner can help you access treatment with priority and make sure you can access safe injecting equipment. | |
| Have you ever reused a needle/syringe or equipment after someone else had used it, including your partner (even if it was cleaned) | **No, Never**  **Not in the past 3 months**  **Yes, within the past 3 months** |
| Why are we asking this?  Sharing injecting equipment is the most common way you can be infected with hepatitis C. You can also be re-infected after treatment.  Your healthcare practitioner can help make sure you have continued access to clean injecting equipment and if you do get re-infected we can make sure you always have access to testing and treatment. | |
| Have you been prescribed methadone, buprenorphine or suboxone? | **Yes, am on it now**  **No, but I might want to start it in the future**  **No** |
| Why are we asking this?  Knowing that you are on opioid substitution therapy is helpful because there is an option to have your hepatitis C medications dispensed at the same time as your opioid substitution therapy.  If you are interested in the taking it in the future, your healthcare practitioner can give you more information and a referral to a local service. | |
| Will you experience any life disruptions in the next 6/9 months? | **No**  **Moving house**  **Going overseas/interstate**  **Major medical procedure**  **Potential imprisonment**  **Pregnancy**  **Other_______________________** |
| Why are we asking this?  Most hepatitis C treatments take between 8 – 12 weeks. You also need to have blood tests at the end of your treatment and 3 months later.  Taking your medicines every day without any disruptions is very important and will give you the best opportunity to be cured. If there are any disruptions, your healthcare practitioner can help you make sure your treatments and tests can be completed. | |
